# Modeling Measles Epidemics

**DOI:** 10.1371/journal.pmed.0020384

**Published:** 2005-10-18

Measles is still a leading cause of death among young children, despite the availability of an effective vaccine for the past 40 years. Although it is rare in many developed countries, it remains a common illness in many developing countries, and more than half a million people, mostly children, died from measles in 2003. There is a global effort to reduce measles mortality, which aims to achieve routine vaccination coverage of at least 90% of people. In countries with high vaccination coverage, the epidemic pattern has changed from a roughly biennial cycle to an irregular sequence of outbreaks—mainly from imported cases and people refusing vaccination.

A proper understanding of the size and timing of these outbreaks is crucial for monitoring a vaccination program and assessing the risk of future measles outbreaks. Previous studies have shown that the size of an outbreak depends on the fraction of susceptible individuals in a population but also on chance events in the transmission process. These previous studies presupposed the existence of a critical threshold level for the fraction of susceptible individuals, below which introduction of infection will lead to only minor outbreaks. The probability that the outbreak will be major increases with the fraction of susceptible individuals in excess of the threshold.

In a *PLoS Medicine* paper, Jacco Wallinga and colleagues stress the need for direct observations that show the relationship between fraction of susceptible individuals and infection attack rate during irregular measles outbreaks in highly vaccinated populations. Such observations could shed light on how health authorities should interpret infection attack rates and assess the risk of future measles outbreaks, they say. [Fig pmed-0020384-g001]

**Figure pmed-0020384-g001:**
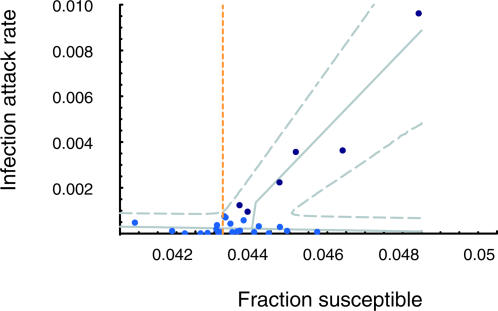
Relation between fraction of susceptible individuals and attack rate

To overcome this lack of information, the team investigated measles outbreaks over 28 years in a Dutch population. They estimated the fraction of susceptible individuals and infection attack rates for each epidemic year during the study period. The average vaccine coverage during this period was 93%, and there was no sustained measles transmission. Several measles outbreaks occurred in communities with low vaccine coverage, but these ended without intervention. The team found a clear threshold value for the fraction of susceptible individuals below which only minor outbreaks occurred, and above which both minor and major outbreaks occurred. They suggest that a precise quantitative relationship exists between the fraction of susceptible individuals in excess of this threshold and the infection attack rate during major outbreaks: one additional unvaccinated person is associated with more than one—almost two—infected persons in subsequent major outbreaks.

These findings update previous thinking on measles vaccination strategies. Previous approaches relied on a statistical description of outbreak sizes based on the theory of solid herd immunity in a homogeneous population. However, the authors' observations are suggestive of occasional, localized lapses in herd immunity, which would allow for major outbreaks within communities with low vaccine coverage. The authors suggest that major outbreaks can be averted only if additional vaccination programs, first tested in mathematical models, target communities at risk, or if measles cases are prevented from entering those communities.

The authors warn that great care should be taken in interpreting a time series of measles case notifications without a corresponding time series of the fraction of susceptible individuals. A few measles cases over several years might not necessarily signal that solid herd immunity has been achieved. It might in fact indicate an increasing risk of a future major measles outbreak. Therefore, these findings could also be used to improve the design of disease-surveillance systems by including monitoring of the changes in fraction of susceptible individuals through time. And whenever the transmission chain is interrupted, the estimated change in fraction of susceptible individuals could be used to assess the risk of future major measles outbreaks.

